# Effect of High, Medium, and Low Molecular Weight Hyaluronan on Inflammation and Oxidative Stress in an* In Vitro* Model of Human Nasal Epithelial Cells

**DOI:** 10.1155/2016/8727289

**Published:** 2016-04-24

**Authors:** Giusy Daniela Albano, Anna Bonanno, Luca Cavalieri, Eleonora Ingrassia, Caterina Di Sano, Liboria Siena, Loredana Riccobono, Rosalia Gagliardo, Mirella Profita

**Affiliations:** ^1^Institute of Biomedicine and Molecular Immunology “A. Monroy” (IBIM), National Research Council of Italy (CNR), Via Ugo La Malfa 153, 90146 Palermo, Italy; ^2^Chiesi Farmaceutici S.p.A, Parma, Italy

## Abstract

IL-17A is involved in the activation of oxidative stress and inflammation in nasal epithelial cells. Hyaluronan (HA) in its high molecular weight form (HMW-HA) shows anti-inflammatory responses in contrast to low and medium molecular weight HA (LMW-HA and MMW-HA). The aim of this study was to investigate the pro- or anti-inflammatory biologic function of HA at different molecular weight in an* in vitro* model of nasal inflammation IL-17A mediated. We evaluated the ERK1/2 and I*κ*B*α* phosphorylation, NF-*κ*B signal pathway activation, ROS production, IL-8 and NOX-4 protein, and mRNA levels, in nasal epithelial cells RPMI 2650 stimulated with recombinant human (rh) IL-17A. Furthermore, the cells were treated with HMW-HA, MMW-HA, LMW-HA, and U0126. Our results showed that rhIL-17A increased the ERK1/2, I*κ*B*α* phosphorylation and NF-*κ*B signal pathway activation, ROS production, IL-8 and NOX-4 proteins, and mRNA levels. The addiction of HMW-HA or U0126 showed a significant downregulatory effect on inflammation due to the rhIL-17A stimulation in nasal epithelial cells. IL-17A is able to generate oxidative stress and inflammation via the activation of ERK1/2/NF-*κ*B pathway in nasal epithelial cells. The HMW-HA might represent a coadjuvant of the classic anti-inflammatory/antioxidative treatment of nasal epithelial cells during IL-17A nasal inflammation.

## 1. Introduction

Allergic rhinitis is an extremely common medical problem characterized by nasal congestion, clear rhinorrhea, sneezing, and itching. The presence of an uncontrolled inflammation in the upper airways may compromise the control of allergic rhinitis with a consequent progression of the diseases [1]. IL-17A and oxidative stress are involved in the development and progression of allergic rhinitis by the activation of nasal epithelial cells [2].

Hyaluronan (HA) is a glycosaminoglycan widely distributed in tissues and is a normal constituent of airway secretions [3, 4]. It is a major component of the extracellular matrix and it plays a key role in regulating inflammation that is associated with accumulation and turnover of HA polymers by multiple cell types. Increasingly through the years, HA has become recognized as an active participant in inflammatory, angiogenic, fibrotic, and cancer promoting processes [5].

In the airways, HA is produced by submucosal glands and by superficial airway epithelial cells [6, 7]. It is synthesized by hyaluronan synthases (HAS) at the plasma membrane and normally released, as a high molecular weight polymer (HMW) (>1000 kDa), into the extracellular milieu [8, 9]. Low molecular weight HA (LMW, defined as <500 kDa) can be produced by the breakdown of HMW-HA to LMW-HA, or by* de novo* synthesis by hyaluronan synthases during inflammatory process [10, 11]. The differential activities of HA and its degradation products are due, in part, to regulation of multiple HA-binding proteins, including cluster of differentiation 44 (CD44) [10].

Most HA functions have been shown to be size-dependent: the HMW molecules have been reported to exert anti-inflammatory and immunosuppressive effects, while LMW stimulate gene expression and synthesis of proinflammatory protein such as cytokines and chemokines [10, 12]. These results strongly support the role of HA and HA-binding proteins in lung pathobiology of asthma [13, 14]. HA appears in low concentrations in bronchoalveolar lavage fluid (BAL) from healthy individuals and is elevated in BAL of asthma patients [15, 16]. The concentration of HA in BAL was found to significantly correlate with the severity of asthma [17]. However, the role of HA homeostasis in human asthma and allergic rhinitis has not been thoroughly explored.* In vitro* studies showed that LMW-HA induces, via ERK1/2 and NF-*κ*B signaling, the production of IL-8 in transformed bronchial epithelial cells [18], suggesting that LMW-HA is able to play a role in acute lung inflammation. However, there were no studies showing the role of LMW-HA in nasal inflammation during allergic rhinitis.

HA is an endogenous compound having an important role in mucociliary clearance and mucosal surface healing and repair of nasal epithelial cells [19]. It was observed that the addition of sodium HA to intranasal corticosteroid and systemic antihistamine reduced the neutrophil count seen on nasal cytology in patients with allergic and nonallergic rhinitis and improved several clinical and endoscopic parameters while being well tolerated. Furthermore, the use of intranasal sodium HA in patients undergoing functional endoscopic sinus surgery for nasal polyposis augmented the improvement in mucociliary clearance observed following this procedure and improved several clinical and endoscopic parameters [19, 20]. These data provide encouraging evidence of the beneficial effects of sodium HA in the care of patients with altered function of nasal epithelial cells and suggest its potential involvement in the control of nasal inflammation and oxidative stress.

We aimed to perform a study to test the use of LMW-HA (500 kDa), MMW-HA (~900 kDa), and HMW-HA (~1600 kDa) in an* in vitro* model of oxidative stress (ROS production and NOX-4 expression) and inflammation (IL-8 synthesis) generated by rhIL-17A in nasal epithelial cells. This study might be appropriate to identify the potential therapeutic application of HMW-HA as coadjuvant of the classic anti-inflammatory treatment in the pathological inflammation and oxidative stress generated in nasal epithelium during the chronic inflammation of the airways.

## 2. Materials and Methods

### 2.1. Nasal Epithelial Cell Cultures

RPMI 2650 cell lines (ATCC-CCL-30) were purchased from American Type Culture Collection (ATCC; Rockville, MD, USA) and supplied at Passage 26. This line represents an appropriate* in vitro* nasal model able to grow a polarized epithelium resembling nasal mucosa [21]. Cells were cultured in complete culture medium (MEM minimum essential media containing 10% FCS, L-glutamine 2 mM, gentamicin 50 mg/mL, MEM NEAA 0.5%, and sodium pyruvate 1 nM).

### 2.2. Stimulation of RPMI 2650 Cells

The cells were seeded in standard six-well culture plates in MEM 10% FCS and grown to 60–70% confluence prior to treatment. RPMI 2650 cells were stimulated with recombinant human IL-17A (rhIL-17A) (R&D Systems, Minneapolis, MN) (20 ng/mL) as previously described [2].

To determine the role of the MAPK pathways in the activation of oxidative stress and IL-8 production, RPMI 2650 cells were stimulated with rhIL-17A for 30 min, 6 hrs, or 18 hrs in the presence or absence of inhibitor U0126 (1,4-diamino-2,3-dicyano-1,4-bis(o-aminophenylmercapto) butadiene monoethanolate) (25 *μ*M) (Sigma-Aldrich s.r.l., Milan, Italy) (specific inhibitor of MEK1 and MEK2 MAP kinase kinase; MAPKK). The RPMI 2650 cells were stimulated with phorbol 12-myristate 13-acetate (PMA, 50 ng/mL) as positive control for ERK1/2 (extracellular-signal-regulated kinases) and I*κ*B*α* (nuclear factor of kappa light polypeptide gene enhancer in B-cells inhibitor, alpha) activation.

To test the activity of different HA molecular weight on the oxidative stress and IL-8 production, RPMI 2650 cells were stimulated with rhIL-17A for 30 min, 6 hrs, or 18 hrs in the presence or absence of HMW-HA (1600 kD, IALUCLENNY, 0.12%, Chiesi Farmaceutici S.p.A.) (100 *μ*g/mL) or MMW-HA (900 kDa, YABRO, 0.3%, Ibsa Farmaceutici Italia) (100 *μ*g/mL) or LMW-HA (370 kDa, cod. H7630, Sigma Chemical Co. St. Louis, MO) (100 *μ*g/mL). To confirm the specific action of HA, HMW-HA samples were degraded with* Streptomyces* hyaluronidase (HAdase, catalyzing the random hydrolysis of HA) (0.05 Units/2 *μ*g) (Sigma Chemical Co., St. Louis, MO) at 60°C for 72 hours followed by 10 minutes at 65°C.

### 2.3. ERK1/2, pI*κ*B*α*, and NF-*κ*B Activation

The effect of rhIL-17A on ERK1/2 and I*κ*B*α* activation was evaluated in RPMI 2650 cells stimulated for 30 minutes. We performed western blot analysis in total cell lysates and nuclear extracts. In total extract, we studied pERK1/2 using an anti-phospho ERK1/2 rabbit monoclonal antibody, pI*κ*B*α* using an anti-phospho I*κ*B*α* rabbit antibody (Cell Signaling Technology, Beverly, MA), and anti-*β*-actin (Sigma St. Louis, MO). In nuclear extracts, we studied the nuclear translocation of NF-*κ*B, using an anti NF-*κ*B p65 (C-20) antibody (Santa Cruz Biotechnology, Inc., MI, Italy). Nuclear extracts were obtained by NE-PER Nuclear and Cytoplasmic Extraction Kit (Thermo Scientific), providing an efficient cell lysis and separate cytoplasmic and nuclear protein fractions by centrifugation. Additionally, we evaluated ERK1/2 and NF-*κ*B activation using two commercially available ELISA kits (SuperArray Bioscience, Frederick, MD) that measure phosphorylated and total ERK1/2, as well as phosphorylated and total NF-*κ*B. Results are expressed, respectively, as pERK1/2/tERK1/2 ratio and pNF-*κ*B/tNF-*κ*B ratio and normalized to protein content.

### 2.4. Detection of Intracellular ROS

The intracellular reactive oxygen species (ROS) generation was evaluated in RPMI 2650 stimulated with rhIL-17A (20 ng/mL) for 6 hrs. The cells were trypsinised, washed in PBS, collected in FACS tubes, and then incubated with 1 mM of the oxidant-sensitive dye 2,7-dichlorofluorescein diacetate (DCFH-DA) in PBS for 10 min in the dark at room temperature. After washing, cells were suspended in PBS and then analysed by flow cytometry for fluorescence positive cells using a FACSCalibur*™* flow cytometer (Becton Dickinson, Mountain View, CA, USA). Negative controls consisted of RPMI 2650 cells cultured without DCFH-DA. Gating on the cells, excluding debris, was performed using forwards and sideways scatter patterns.

### 2.5. Detection of NOX-4 and IL-8 by Western Blot

NOX-4 (NADPH oxidase) and IL-8 were evaluated in RPMI 2650 stimulated with rhIL-17A for 18 hrs in the presence or absence of HAs. Total proteins were extracted from stimulated RPMI 2650 cells using a lysis buffer (NaCl 50 mM, Tris-HCl 10 mM, EDTA 5 mM, and NP-40 1%) containing protease and phosphatase inhibitors. Protein concentration was assessed using the Bradford method. The total protein extracts were separated by SDS-PAGE on 10% gradient gels followed by electroblotting onto nitrocellulose membranes. Western blot was performed using a primary rabbit polyclonal anti-NOX-4 (H-330, Santa Cruz Biotechnology, Inc., Santa Cruz, CA) and a mouse monoclonal anti-IL-8 (B-2, Santa Cruz Biotechnology, Inc., Santa Cruz, CA). *β*-actin (Sigma-Aldrich) was used as a housekeeping protein to control the total amount of protein in each sample. Primary antisera were visualized with horseradish peroxidase-conjugated secondary antibody (Sigma-Aldrich, St. Louis, MO) and developed with an enhanced chemiluminescence system (GE Healthcare, Chalfont St. Giles, UK). Approximate molecular masses were determined using calibrated prestained standards (GE Healthcare).

### 2.6. Quantitative Real-Time Reverse Transcription-Polymerase Chain Reaction (RT-PCR) of NOX-4 and IL-8

Total RNA was extracted from RPMI 2650 cells with TRIzol Reagent (Invitrogen) following the manufacturer's instructions and was reverse-transcribed into cDNA, using M-MLV-RT and oligo (dT)_12-18_ primer (Invitrogen). Quantitative real-time PCR of NOX-4 and IL-8 transcripts was carried out on StepOne Plus Real-Time PCR System (Applied Biosystems, Foster City, CA, USA) using specific FAM-labeled probe and primers (prevalidated TaqMan Gene Expression Assay for NOX-4, Hs00418356m1, and IL-8, Hs00174103m1; Assays on Demand, Applied Biosystems). NOX-4 and IL-8 gene expression were normalized to glyceraldehyde-3-phosphate dehydrogenase (GAPDH) endogenous control gene. Relative quantitation of gene expression was carried out with the comparative C_T_ method (2^−ΔΔCt^) and was plotted as fold change compared to untreated cells chosen as the reference sample.

### 2.7. Gel Image Evaluation

Gel images were taken with an EPSON GT-6000 scanner and then imported into a National Institutes of Health Image analysis 1.61 program to determine band intensities. Data are expressed as arbitrary densitometric units corrected against the density of *β*-actin bands.

### 2.8. Statistics

We tested normal distribution of the data with Kolmogorov–Smirnov test. Analysis of variance (ANOVA) corrected with Fisher's test and *t*-test were used for comparisons. Data are expressed as mean ± standard deviation (SD). *p* < 0.05 was accepted as statistically significant.

## 3. Results

### 3.1. ERK1/2 and I*κ*B*α* Activation in RPMI 2650 Stimulated with rhIL-17A

**Figure 1:**
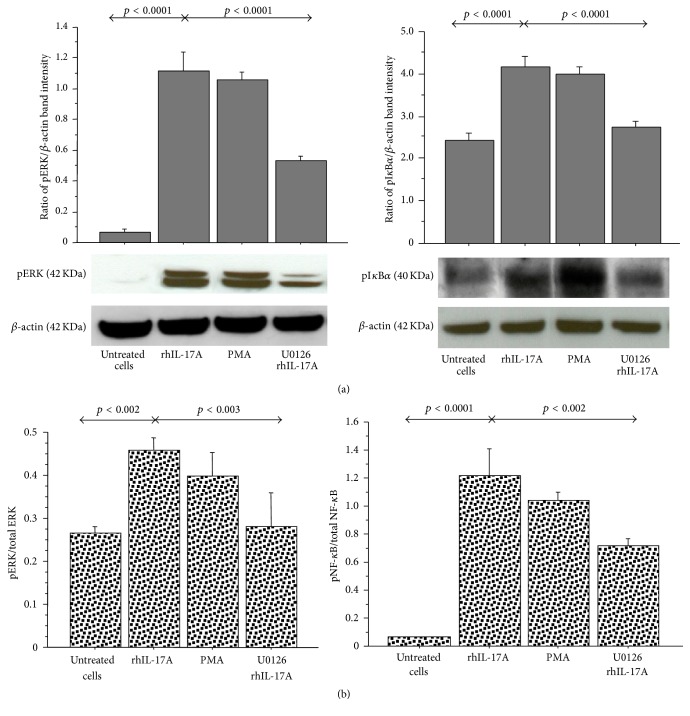
Effect of U0126 inhibitor on ERK and I*κ*B*α* phosphorylation in RPMI 2650 cells stimulated with rhIL-17A. The cells were stimulated with rhIL-17A (20 ng/mL) or PMA (50 ng/mL) for 30 min in absence or presence of U0126 (25 *μ*M). (a) pERK and pI*κ*B*α* protein expression were evaluated in the cell lysates by western blot. The results were expressed as ratio of band intensity and *β*-actin of 3 separate experiments. Representative gel images of pERK, pI*κ*B*α*, and *β*-actin are shown. (b) The activation of ERK1/2 and NF-*κ*B for each experimental condition was tested for the pERK1/2/total ERK1/2 ratio and for the pNF-*κ*B/total NF-*κ*B, respectively, by ELISA and normalized for protein content. ANOVA with Fisher's test correction was used for the analysis of the data. *p* < 0.05 was statistically significant.

The stimulation of RPMI 2650 with rhIL-17A for 30 minutes significantly increased pERK1/2 and pI*κ*B*α* activation in comparison to untreated cells (*p* < 0.0001) by western blot analysis. The preincubation of the cells with U0126 (25 *μ*M) significantly decreased the levels of pERK1/2 and pI*κ*B*α*, as observed in the cells stimulated with rhIL-17A (*p* < 0.0001) (Figure 1(a)). Accordingly, the levels of pERK1/2/total ERK1/2 ratio and pNF-*κ*B/total NF-*κ*B ratio significantly increased in the cells stimulated with rhIL-17A (*p* < 0.002 and *p* < 0.0001, resp.) and showed a statistically significant decrease when the cells were preincubated with U0126 (*p* < 0.003 and *p* < 0.002 resp.) (Figure 1(b)).

### 3.2. ROS Production, NOX-4, and IL-8 Proteins in RPMI 2650 Stimulated with rhIL-17A

**Figure 2:**
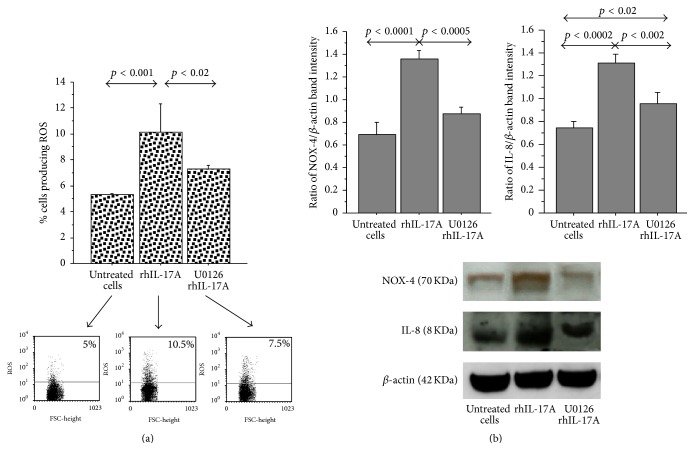
Effect of U0126 inhibitor in RPMI 2650 cells stimulated with rhIL-17A. (a) The cells were stimulated with rhIL-17A (20 ng/mL) for 6 hrs in absence or presence of U0126 (25 *μ*M). ROS production was evaluated in the cells by flow cytometry. The bars represent the mean ± SD of 3 separate experiments. Representative flow cytometry are shown; (b) the cells were stimulated with rhIL-17A (20 ng/mL) for 18 hrs in absence or presence of U0126 (25 *μ*M). NOX-4 and IL-8 protein expression were evaluated in the cell lysates by western blot. The results were expressed as ratio of band intensity and *β*-actin of 3 separate experiments. Representative western blot is shown. ANOVA with Fisher's test correction was used for the analysis of the data. *p* < 0.05 was statistically significant.

The ROS production showed a significant increase in RPMI 2650 cells stimulated for 6 hrs with rhIL-17A (20 ng/mL) (*p* < 0.001), compared to untreated cells. The pretreatment of the cells with U0126 (25 *μ*M) significantly decreased ROS production in RPMI 2650 cells stimulated with rhIL-17A (*p* < 0.02) (Figure 2(a)).

NOX-4 and IL-8 protein significantly increased in RPMI 2650 cells stimulated for 18 hrs with rhIL-17A (20 ng/mL) (*p* < 0.0001 and *p* < 0.0002), in comparison to untreated cells. The preincubation of RPMI 2650 cells with U0126 (25 *μ*M) significantly decreased NOX-4 (*p* < 0.0005) and IL-8 production (*p* < 0.002) in nasal epithelial cells stimulated with rhIL-17A (Figure 2(b)).

### 3.3. Effect of HMW-HA, MMW-HA, and LMW-HA on ERK1/2 and NF-*κ*B Activation

**Figure 3:**
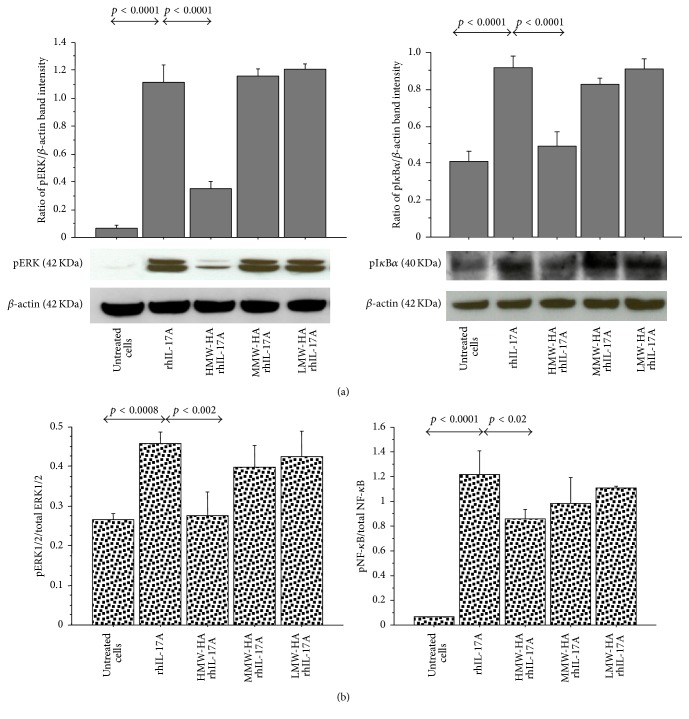
Effect of HMW-HA, MMW-HA, and LMW-HA on ERK1/2 and NF-*κ*B signal pathway in RPMI 2650 cells stimulated with rhIL-17A. The cells were preincubated with HMW-HA (100 *μ*g/mL), MMW-HA (100 *μ*g/mL), and LMW-HA (100 *μ*g/mL) for 1 h and then stimulated with rhIL-17A (20 ng/mL) for 30 min; (a) pERK and pI*κ*B*α* protein expression were evaluated in the cell lysates by western blot. The bars represent the ratio of band intensity and *β*-actin of 3 separate experiments. Representative gel images of pERK, pI*κ*B*α*, and *β*-actin are shown; (b) the activation of ERK1/2 and NF-*κ*B for each experimental condition was tested for the pERK1/2/total ERK1/2 ratio and pNF-*κ*B/total NF-*κ*B ratio by ELISA and normalized for protein content. ANOVA with Fisher's test correction was used for the analysis of the data. *p* < 0.05 was statistically significant.

The pretreatment of the RPMI 2650 with HMW-HA significantly inhibited the levels of pERK1/2 (*p* < 0.0001) and pI*κ*B*α* (*p* < 0.0001) in the cells stimulated with rhIL-17A, compared to the cells treated with rhIL-17A alone (Figure 3(a)). Accordingly, the pretreatment of the RPMI 2650 with HMW-HA significantly reduced the pERK/total ERK ratio and pNF-*κ*B/total NF-*κ*B ratio in the cells stimulated with rhIL-17A compared to the cells treated with rhIL-17A alone (*p* < 0.002, *p* < 0.02, resp.) (Figure 3(b)). Conversely, the pretreatment of the RPMI 2650 with MMW-HA and LMW-HA did not control the activity of rhIL-17A on ERK1/2 and NF-*κ*B pathway activation.

### 3.4. Effect of HMW-HA, MMW-HA, and LMW-HA on ROS Production, NOX-4, and IL-8 Synthesis

**Figure 4:**
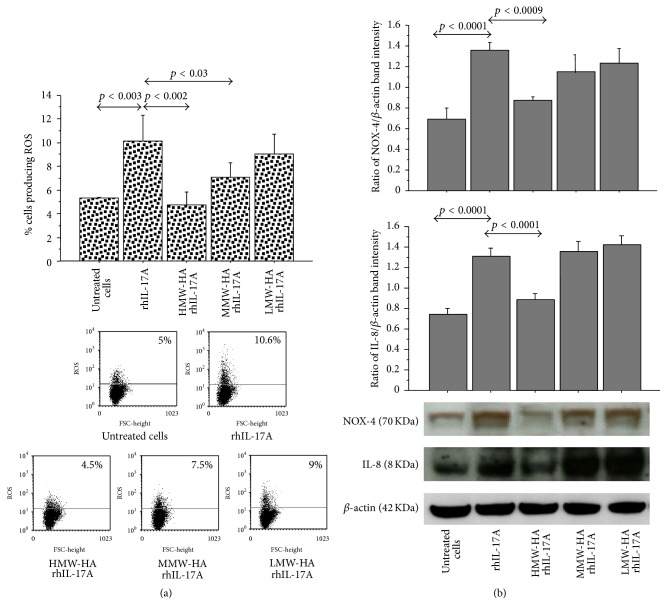
Effect of HMW-HA, MMW-HA, and LMW-HA in RPMI 2650 cells stimulated with rhIL-17A. (a) The cells were preincubated with HMW-HA (100 *μ*g/mL), MMW-HA (100 *μ*g/mL), and LMW-HA (100 *μ*g/mL) for 1 h and then stimulated with rhIL-17A (20 ng/mL) for 6 hrs. ROS production was evaluated in the cells by flow cytometry. The bars expressed mean ± SD of 3 separate experiments. Representative flow cytometry is shown; (b) the cells were preincubated with HMW-HA (100 *μ*g/mL), MMW-HA (100 *μ*g/mL), and LMW-HA (100 *μ*g/mL) for 1 h and then stimulated with rhIL-17A (20 ng/mL) for 18 hrs. NOX-4 and IL-8 protein synthesis were evaluated in the cell lysates by western blot. The bars represent the ratio of band intensity and *β*-actin of 3 separate experiments. Representative western blot is shown. ANOVA with Fisher's test correction was used for the analysis of the data. *p* < 0.05 was statistically significant.

The pretreatment of RPMI 2650 with HMW-HA and MMW-HA reduced the induction of ROS production in the cells stimulated with rhIL-17A compared to the cells treated with rhIL-17A alone (*p* < 0.002 and *p* < 0.03, resp.). Conversely, the pretreatment of the cells with LMW-HA did not affect the ROS production generated by rhIL-17A stimulation in RPMI 2650 (Figure 4(a)).

The pretreatment of RPMI 2650 cells with HMW-HA significantly inhibited NOX-4 and IL-8 synthesis (*p* < 0.0009, *p* < 0.0001, resp.) in the cells stimulated with rhIL-17A compared to the cells treated with rhIL-17A alone (Figure 4(b)). Conversely, the pretreatment of the RPMI 2650 with MMW-HA and LMW-HA did not control the activity of rhIL-17A on NOX-4 and IL-8 synthesis.

### 3.5. Effect of U0126, HMW-HA, MMW-HA, and LMW-HA on Nuclear NF-*κ*B Translocation

**Figure 5:**
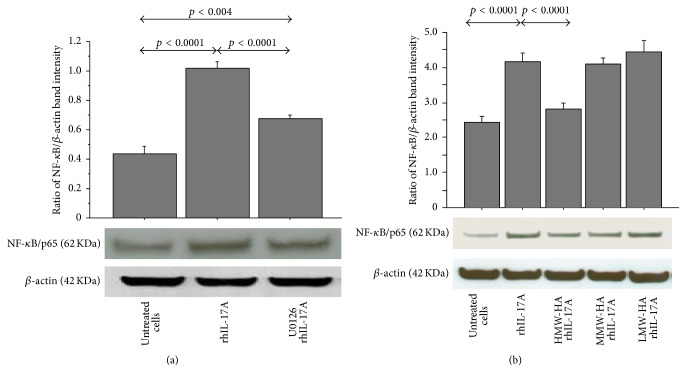
Effect of U0126, HMW-HA, MMW-HA, and LMW-HA on nuclear translocation of NF-*κ*B in RPMI 2650 cells stimulated with rhIL-17A. The cells were preincubated (a) with U0126 (25 *μ*M) or (b) with HMW-HA (100 *μ*g/mL), MMW-HA (100 *μ*g/mL), and LMW-HA (100 *μ*g/mL) for 1 h and then stimulated with rhIL-17A (20 ng/mL) for 30 min; NF-*κ*B was evaluated in nuclear cell lysate by western blot. The bars represent ratio of band intensity and *β*-actin of 3 separate experiments. Representative gel images of NF-*κ*B and *β*-actin are shown. ANOVA with Fisher's test correction was used for the analysis of the data. *p* < 0.05 was statistically significant.

The stimulation of RPMI 2650 with rhIL-17A for 30 minutes significantly increased the nuclear translocation of NF-*κ*B in comparison to untreated cells (*p* < 0.0001) that was significantly decreased when the cells were preincubated with U0126 (25 *μ*M) (*p* < 0.0001) (Figure 5(a)). The pretreatment of RPMI 2650 cells with HMW-HA significantly inhibited the nuclear levels of NF-*κ*B (*p* < 0.0001) in the cells stimulated with rhIL-17A compared to the cells treated with rhIL-17A alone. Conversely, the pretreatment of the RPMI 2650 with MMW-HA and LMW-HA did not control the activity of rhIL-17A on nuclear translocation of NF-*κ*B (Figure 5(b)).

### 3.6. Effect of U0126, HMW-HA, MMW-HA, and LMW-HA on NOX-4 and IL-8 mRNA

**Figure 6:**
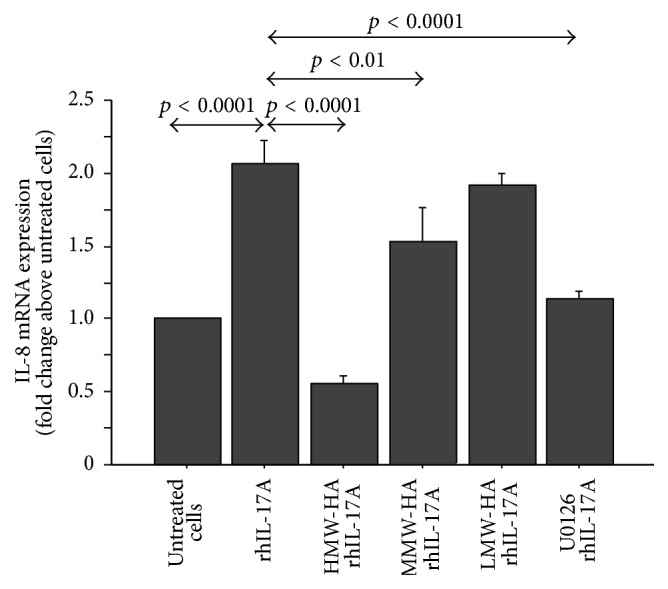
Effect of HMW-HA, MMW-HA, LMW-HA, and U0126 on IL-8 mRNA transcript. The cells were preincubated with HMW-HA (100 *μ*g/mL), MMW-HA (100 *μ*g/mL), LMW-HA (100 *μ*g/mL), and U0126 (25 *μ*M) for 1 h and then stimulated with rhIL-17A (20 ng/mL) for 18 hrs to study IL-8 mRNA. mRNA levels were quantified by quantitative Real-Time PCR (see Materials and Methods for details). The bars represent the mean ± SD of 3 separate experiments. ANOVA with Fisher's test correction was used for the analysis of the data. *p* < 0.05 was statistically significant.

Despite the data on the expression of NOX-4 protein, we did not observe modification of NOX-4 mRNA transcript in the cells stimulated with rhIL-17A for 2, 6, and 18 hrs in the presence or absence of HAs (data not shown). The stimulation of RPMI 2650 with rhIL-17A for 18 hrs significantly increased the levels of IL-8 mRNA transcript in comparison to untreated cells (*p* < 0.0001). The pretreatment of RPMI 2650 with U0126 (25 *μ*M) significantly decreased the levels of IL-8 mRNA in the cells stimulated with rhIL-17A compared to the cells stimulated with rhIL-17A alone (*p* < 0.0001). Furthermore, the pretreatment of RPMI 2650 with HMW-HA (*p* < 0.0001) and MMW-HA (*p* < 0.01)significantly decreased the levels of IL-8 mRNA in the cells stimulated with rhIL-17A compared to the cells stimulated with rhIL-17A alone. Conversely, the pretreatment of the RPMI 2650 with LMW-HA did not control the activity of rhIL-17A on the levels of IL-8 mRNA (Figure 6).

### 3.7. Effect of HAdase HMW-HA on ROS Production, NOX-4, and IL-8 Synthesis

**Figure 7:**
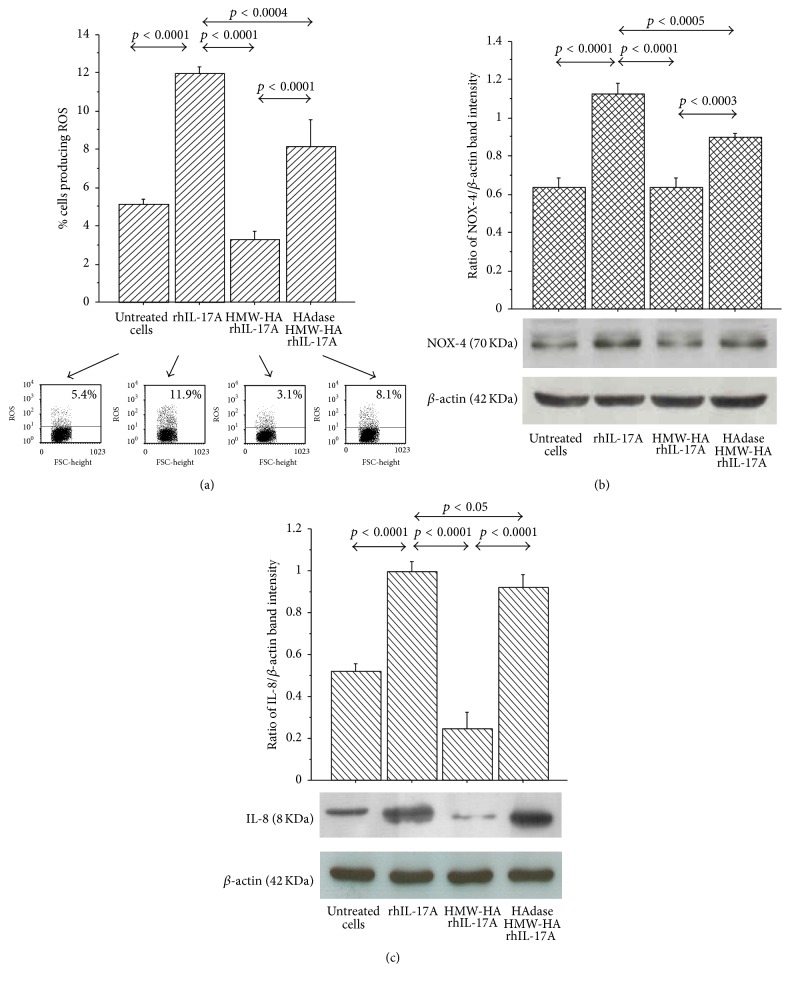
Specific effect of HMW-HA in RPMI 2650 cells stimulated with rhIL-17A. HMW-HA was incubated with HAdase 2.5 units/100 *μ*g (60°C for 72 hrs plus 65°C for 10 min). The cells were preincubated with HMW-HA (100 *μ*g/mL) or HAdase treated HMW-HA for 1 h and stimulated with rhIL-17A (20 ng/mL) for 6 hrs for ROS or 18 hrs for NOX-4 and IL-8. (a) ROS production was evaluated in the cells by flow cytometry. Representative flow cytometry is shown. The results were expressed as the mean ± SD of 3 separate experiments; (b-c) NOX-4 and IL-8 protein synthesis were evaluated in the cell lysates by western blot. The bars represent ratio of band intensity and *β*-actin of 3 separate experiments. Representative western blot is shown. ANOVA with Fisher's test correction was used for the analysis of the data. *p* < 0.05 was statistically significant.

ROS production was significantly increased in RPMI 2650 treated with HMW-HA digested with HAdase (HAdase HMW-HA) in comparison to RPMI 2650 treated with HMW-HA before the stimulation with rhIL-17A for 6 hrs (Figure 7(a)). NOX-4 and IL-8 synthesis were significantly increased in RPMI 2650 treated with HMW-HA digested with HAdase (HAdase HMW-HA) in comparison to RPMI 2650 treated with HMW-HA before the stimulation with rhIL-17A for 18 hrs (Figures 7(b) and 7(c)). However, we underlined that RPMI 2650 treated with rhIL-17A and HAdase HMW-HA showed statistically significant lower levels of ROS, NOX-4, and IL-8 than the cells treated with rhIL-17A alone (Figures 7(a), 7(b), and 7(c)).

## 4. Discussion

**Figure 8:**
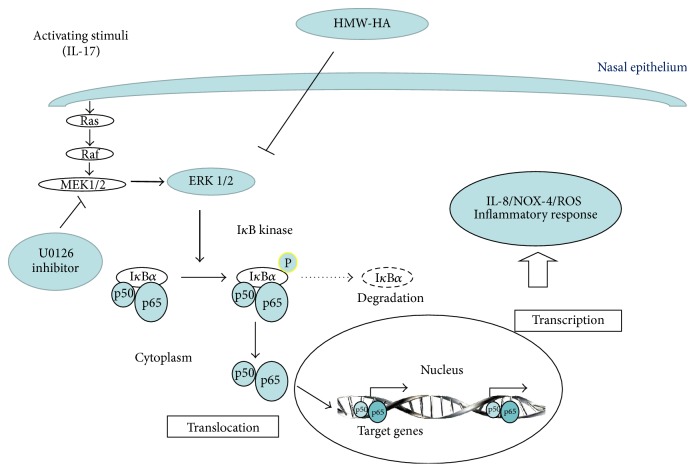
Graphical abstract of the study.

This study suggests the potential role of HMW-HA rather than MMW-HA as coadjuvant of the classic anti-inflammatory treatment during the nasal inflammatory and oxidative process IL-17A mediated. Indeed, our current findings identified the potential ancillary role of HMW-HA in the regulation of oxidative stress (ROS, NOX-4) and IL-8 synthesis generated by epithelial cells during nasal inflammation. Particularly in our* in vitro* model, we identify that HMW-HA might be able to control the mechanism of oxidative stress and inflammation blocking the ERK1/2 intracellular signal pathway activation involved in the NF-*κ*B transcriptional mechanism regulation (Figure 8: graphical abstract).

IL-17 cytokines promote tissue inflammation via the induction of other proinflammatory cytokines and chemokines. Moreover, several studies in humans have demonstrated that Th17 immunity is involved in the pathogenesis of allergic diseases [22] with a potential role in the severity of the disease [23, 24]. The activity of IL-17A is mediated by IL-17 receptor (IL-17R) expressed by both blood cells and structural cells including T-cells and the airway epithelial cells [25]. In this scenario, IL-17A in allergic rhinitis and asthma may cover both the innate and the adaptive aspects representing the crucial crosstalk between immune system and structural cells such as fibroblasts and airway epithelial cells [26, 27]. On the other hand, higher levels of IL-17A were observed in the nasal wash from children with allergic rhinitis compared to healthy control [2], suggesting the relevant action of IL-17A in the nasal inflammation.

RPMI 2650 cell line used in this study has been shown to closely resemble normal human upper airway epithelium with respect to its karyotype, cytokeratin expression, and the presence of mucoid material on the cell surface and was previously used to study interactions of the airway epithelium with cytokines and allergens [21, 28, 29]. The use of RPMI 2650 nasal epithelial cells in the current study reflects our intent to understand the molecular and signaling underpinnings of the activity of IL-17A on nasal epithelial cells and whether the HMW-HA can protect the nasal epithelium from IL-17A mediated inflammation.

Nuclear factor- (NF-) *κ*B, which consists of p50 and p65 subunits, is pivotal in the regulation of many genes including cytokines, chemokines, and adhesion molecules. Activation of NF-*κ*B is dependent on the phosphorylation and degradation of I*κ*B, an endogenous inhibitor that binds to NF-*κ*B in the cytoplasm. The released NF-*κ*B then translocates to the nucleus where it binds to specific NF-*κ*B DNA binding sites and initiates gene expression. NF-*κ*B activates gene expression from NF-*κ*B sites in association with the transactivation domains located in the carboxyl-terminus of the p65 protein [30, 31]. ERK1/2 and NF-*κ*B play an important role in IL-17A-induced cytokine in human airway smooth muscle cells* in vitro* [32]. We demonstrated that IL-17A is able to activate ERK1/2 and I*κ*B*α* phosphorylation, together with the nuclear translocation of NF-*κ*B reduced using a specific inhibitor of MEK1 and MEK2 MAP kinase kinase inhibitor U0126 in nasal epithelial cells. These findings might suggest that IL-17A activity generates the NF-*κ*B translocation to the nucleus via MEKK1 signaling cascades, promoting an increase of NF-*κ*B at binding sites of genes involved in nasal epithelial cells activation during airway inflammation.

NOX-4, localized mainly in the epithelial layer, may play an important role in reactive oxygen species production, contributing to the oxidative stress in allergic rhinitis and nasal polyp tissues [33]. NOX family is a key component of the so-called redox signaling system regulating many cellular responses by intracellular ROS content [34]. IL-17A increases oxidative/nitrosative markers, likely via ERK1/2 downstream signaling in bronchial epithelial cells [35]. Accordingly, we found that IL-17A is able to activate ROS production and NOX-4 expression in nasal epithelial cells reduced by the use of the specific inhibitor U0126. These findings might suggest that IL-17A generates endogenous ROS via MEKK1 signaling cascades able to promote NF-*κ*B activity at the binding site of NOX-4 in nasal epithelial cells.

Several* in vitro* studies have shown that recombinant IL-17A is able to induce IL-6, IL-8, granulocyte colony-stimulating factor (GCS-F), nitric oxide (NO), and prostaglandin E2 (PGE2) in airway epithelial cells [36]. RhIL-17A promotes the release of IL-8 in both nasal and bronchial epithelial cells [2] and IL-17A-induced IL-8 production p38, extracellular-signal-related kinase (ERK), and phosphoinositide-3-kinase (PI3K) pathways, and the latter appeared to be involved in IL-17A-induced GC insensitivity [37]. In our study, IL-17A is able to activate IL-8 production in terms of protein and mRNA in nasal epithelial cells that was reduced by the use of the specific inhibitor U0126. In this scenario, our findings might suggest that IL-17A generates IL-8 synthesis via MEKK1 signaling cascades able to promote NF-*κ*B activity at the binding site of NOX-4 in nasal epithelial cells.

Hyaluronan is the main glycosaminoglycan (80%) produced by the respiratory mucosa and represents the main component of the film covering the upper airways. Together with chondroitin sulfate and heparan sulfate, it stratifies on the mucosal epithelial cells. Its topical use increases and improves the healing process, supporting the nasal functions of restoring and the mucosal tropism. Furthermore, the administration of hyaluronan can protect the sinonasal epithelium from inflammatory and surgical damage although there were no clear data about the cellular and molecular mechanism by which it can act [38]. Recent studies revealed increased amounts of total HA and LMW-HA in animal models of acute lung injury [39–42]. Furthermore, LMW-HA can induce the expression of several proinflammatory cytokines, including IL-8 [42–44]. Moreover, HMW-HA can inhibit LPS-activated PI3K/Akt pathway leading to downregulation of NF-*κ*B with diminished IL-6 production through interaction with ICAM-1 in lipopolysaccharide (LPS-) stimulated U937 macrophages [43]. Additionally, HA suppresses advanced glycation end product (AGE-) induced expression of proinflammatory cytokines and NF-*κ*B nuclear translocation in J774 mouse macrophages [44]. ERK1/2 and NF-*κ*B inhibitors significantly abrogate the response to HMW-HA, suggesting an important role for HA in the regulation of epicardial cell fate via activation of MEKK1 signaling cascades [34]. Accordingly with these observations, we demonstrated that HMW-HA, rather than MMW-HA or LMW-HA, is able to downregulate the activation of ERK1/2 and I*κ*B*α* phosphorylation and nuclear translocation of NF-*κ*B as well as the related ROS, IL-8, and NOX-4 protein. These findings suggest that HMW-HA with the involvement of MEKK1 signaling cascades and NF-*κ*B activity in nasal epithelial cells might be able to exercise a protective role in nasal inflammation and did not increase inflammation. In contrast, in our* in vitro* model, LMW-HA or MMW-HA did not affect the biological activity of IL-17A in nasal epithelial cells. Finally, the use of hyaluronidase in the experimental condition, responsible for the HMW-HA fragmentation in nasal epithelial cells culture, reduced the protective action of HMW-HA confirming its specific activity. Additionally, we observed that RPMI 2650 treated with rhIL-17A and HMW-HA digested with HAdase showed statistically significant lower levels of ROS, NOX-4, and IL-8 than the cells treated with rhIL-17A alone. This data suggest a residual activity of HMW-HA in the presence of* Streptomyces* HAdase. This action is in accord with a random endolytic action pattern of* Streptomyces* HAdase due to the presence of some resistant (less susceptible to enzymolysis) sites, ascribed to restricted enzyme access, in the HA polymer [45]. Nevertheless, our findings clearly provide data on a protective effect of HMW-HA. In this scenario, our results clearly support the protective peculiarity of HMW-HA in the control of the nasal epithelial cell activation during the inflammatory process generated by IL-17A. However, further studies might be necessary to better clarify whether the protective activity of HMW-HA is associated with chemical-physical mechanism or with receptor activity in the nasal epithelium. Finally, while IL-8 mRNA transcript reflects the related protein expression, NOX-4 mRNA transcript was not modulated in our study. Accordingly, it was observed that NOX-4 mRNA is regulated at both transcriptional and posttranscriptional levels, and often, the level of NOX-4 mRNA does not accurately reflect NOX-4 protein expression and functions [46].

## 5. Conclusions

This study identified for the first time that HMW-HA is able to downregulate the mechanism of uncontrolled oxidative stress and inflammation typical of upper airway diseases. Indeed, our results provided encouraging evidence to support possible beneficial effects of HMW-HA in the care of patients with altered function of nasal epithelial cells, supporting its potential ancillary role as coadjuvant of the classic anti-inflammatory treatment of the nose. However, additional clinical studies should be performed to assess the usefulness of these observations in clinical practice.

## List of Abbreviations

HMW: High molecular weight
MMW: Medium molecular weight
LMW: Low molecular weight
HA: Hyaluronan
IL-17A: Interleukin-17A
IL-8: Interleukin-8
ROS: Reactive oxygen species
NOX-4: NADPH oxidase
MAPK: Mitogen-activated protein kinase
MAPKK: MAP kinase kinase
NF-*κ*B: nuclear factor kappa-light-chain-enhancer of activated B-cells
ERK1/2: extracellular-signal-regulated kinases
I*κ*B*α*: Nuclear factor of kappa light polypeptide gene enhancer in B-cells inhibitor, alpha
U0126: 1,4-Diamino-2,3-dicyano-1,4-bis(o-aminophenylmercapto) butadiene monoethanolate
PMA: Phorbol 12-myristate 13-acetate
DCFH-DA: Dye 2,7-dichlorofluorescein diacetate
HAdase: Hyaluronidase.

## References

[1] Bourdin A., Gras D., Vachier I., Chanez P. (2009). Upper airway · 1: allergic rhinitis and asthma: united disease through epithelial cells. *Thorax*.

[2] Albano G. D., Di Sano C., Bonanno A. (2013). Th17 immunity in children with allergic asthma and rhinitis: a pharmacological approach. *PLoS ONE*.

[3] Schmekel B., Hörnblad Y., Hvatum M., Norlund A.-L., Venge P. (1995). Kinetic retrieval of eosinophil cationic protein, hyaluronan, secretory IgA, albumin, and urea during BAL in healthy subjects. *Chest*.

[4] Midulla F., Villani A., Merolla R., Bjermer L., Sandstrom T., Ronchetti R. (1995). Bronchoalveolar lavage studies in children without parenchymal lung disease: cellular constituents and protein levels. *Pediatric Pulmonology*.

[5] Petrey A. C., de la Motte C. A. (2014). Hyaluronan, a crucial regulator of inflammation. *Frontiers in Immunology*.

[6] Monzon M. E., Casalino-Matsuda S. M., Forteza R. M. (2006). Identification of glycosaminoglycans in human airway secretions. *American Journal of Respiratory Cell and Molecular Biology*.

[7] Basbaum C. B., Finkbeiner W. E. (1988). Airway secretion: a cell-specific analysis. *Hormone and Metabolic Research*.

[8] Itano N., Kimata K. (2002). Mammalian hyaluronan synthases. *IUBMB Life*.

[9] Weigel P. H., Hascall V. C., Tammi M. (1997). Hyaluronan synthases. *The Journal of Biological Chemistry*.

[10] Liu Y.-Y., Lee C.-H., Dedaj R. (2008). High-molecular-weight hyaluronan—a possible new treatment for sepsis-induced lung injury: a preclinical study in mechanically ventilated rats. *Critical Care*.

[11] Laurent T. C., Fraser J. R., Laurent U. B., Engström-Laurent A. (1995). Hyaluronan in inflammatory joint disease. *Acta Orthopaedica Scandinavica. Supplementum*.

[12] Noble P. W. (2002). Hyaluronan and its catabolic products in tissue injury and repair. *Matrix Biology*.

[13] Lennon F. E., Singleton P. A. (2011). Role of hyaluronan and hyaluronan-binding proteins in lung pathobiology. *American Journal of Physiology—Lung Cellular and Molecular Physiology*.

[14] Liang J., Jiang D., Jung Y. (2011). Role of hyaluronan and hyaluronan-binding proteins in human asthma. *Journal of Allergy and Clinical Immunology*.

[15] Soderberg M., Bjermer L., Hallgren R., Lundgren R. (1989). Increased hyaluronan (hyaluronic acid) levels in bronchoalveolar lavage fluid after histamine inhalation. *International Archives of Allergy and Applied Immunology*.

[16] Vignola A. M., Chanez P., Campbell A. M. (1998). Airway inflammation in mild intermittent and in persistent asthma. *American Journal of Respiratory and Critical Care Medicine*.

[17] Bousquet J., Chanez P., Lacoste J. Y. (1991). Indirect evidence of bronchial inflammation assessed by titration of inflammatory mediators in BAL fluid of patients with asthma. *The Journal of Allergy and Clinical Immunology*.

[18] Ochoa C. D., Garg H. G., Hales C. A., Quinn D. A. (2011). Low molecular weight hyaluronan, via AP-1 and NF-*κ*B signalling, induces IL-8 in transformed bronchial epithelial cells. *Swiss Medical Weekly*.

[19] Gelardi M., Guglielmi A. V. N., De Candia N., Maffezzoni E., Berardi P., Quaranta N. (2013). Effect of sodium hyaluronate on mucociliary clearance after functional endoscopic sinus surgery. *European Annals of Allergy and Clinical Immunology*.

[20] Gelardi M., Iannuzzi L., Quaranta N. (2013). Intranasal sodium hyaluronate on the nasal cytology of patients with allergic and nonallergic rhinitis. *International Forum of Allergy and Rhinology*.

[21] Kreft M. E., Jerman U. D., Lasič E. (2015). The characterization of the human nasal epithelial cell line RPMI 2650 under different culture conditions and their optimization for an appropriate *in vitro* nasal model. *Pharmaceutical Research*.

[22] Zhao J., Yang J., Gao Y., Wei G. (2010). Th17 immunity in patients with allergic asthma. *International Archives of Allergy and Immunology*.

[23] Wang Y. H., Liu Y. J. (2008). The IL-17 cytokine family and their role in allergic inflammation. *Current Opinion in Immunology*.

[24] Wang Y. H., Wills-Karp M. (2011). The potential role of interleukin-17 in severe asthma. *Current Allergy and Asthma Reports*.

[25] Hung L.-Y., Velichko S., Huang F., Thai P., Wu R. (2008). Regulation of airway innate and adaptive immune responses: the IL-17 paradigm. *Critical Reviews in Immunology*.

[26] Ouyang W., Kolls J. K., Zheng Y. (2008). The biological functions of T helper 17 cell effector cytokines in inflammation. *Immunity*.

[27] McKinley L., Alcorn J. F., Peterson A. (2008). TH17 cells mediate steroid-resistant airway inflammation and airway hyperresponsiveness in mice. *The Journal of Immunology*.

[28] Reichl S., Becker K. (2012). Cultivation of RPMI 2650 cells as an in-vitro model for human transmucosal nasal drug absorption studies: optimization of selected culture conditions. *Journal of Pharmacy and Pharmacology*.

[29] Salib R. J., Lau L. C., Howarth P. H. (2005). The novel use of the human nasal epithelial cell line RPMI 2650 as an in vitro model to study the influence of allergens and cytokines on transforming growth factor-*β* gene expression and protein release. *Clinical and Experimental Allergy*.

[30] Baldwin A. S. (1996). The NF-kappa B and I kappa B proteins: new discoveries and insights. *Annual Review of Immunology*.

[31] Finco T. S., Baldwin A. S. (1995). Mechanistic aspects of NF-*κ*B regulation: the emerging role of phosphorylation and proteolysis. *Immunity*.

[32] Wuyts W. A., Vanaudenaerde B. M., Dupont L. J., van Raemdonck D. E., Demedts M. G., Verleden G. M. (2005). Interleukin-17-induced interleukin-8 release in human airway smooth muscle cells: role for mitogen-activated kinases and nuclear factor-*κ*B. *Journal of Heart and Lung Transplantation*.

[33] Moon J. H., Kim T. H., Lee H. M. (2009). Overexpression of the superoxide anion and NADPH oxidase isoforms 1 and 4 (NOX1 and NOX4) in allergic nasal mucosa. *American Journal of Rhinology and Allergy*.

[34] Dröge W. (2002). Free radicals in the physiological control of cell function. *Physiological Reviews*.

[35] Montalbano A. M., Anzalone G., Albano G. D. (2013). Beclomethasone dipropionate and formoterol reduce oxidative/nitrosative stress generated by cigarette smoke extracts and IL-17A in human bronchial epithelial cells. *European Journal of Pharmacology*.

[36] Kawaguchi M., Kokubu F., Kuga H. (2001). Modulation of bronchial epithelial cells by IL-17. *Journal of Allergy and Clinical Immunology*.

[37] Zijlstra G. J., Ten Hacken N. H. T., Hoffmann R. F., van Oosterhout A. J. M., Heijink I. H. (2012). Interleukin-17A induces glucocorticoid insensitivity in human bronchial epithelial cells. *European Respiratory Journal*.

[38] Castelnuovo P., Tajana G., Terranova P., Digilio E., Bignami M., Macchi A. (2015). From modeling to remodeling of upper airways: centrality of hyaluronan (hyaluronic acid). *International Journal of Immunopathology and Pharmacology*.

[39] Bracke K. R., Dentener M. A., Papakonstantinou E. (2010). Enhanced deposition of low-molecular-weight hyaluronan in lungs of cigarette smoke—exposed mice. *American Journal of Respiratory Cell and Molecular Biology*.

[40] Bai K.-J., Spicer A. P., Mascarenhas M. M. (2005). The role of hyaluronan synthase 3 in ventilator-induced lung injury. *American Journal of Respiratory and Critical Care Medicine*.

[41] Nettelbladt O., Hallgren R. (1989). Hyaluronan (hyaluronic acid) in bronchoalveolar lavage fluid during the development of bleomycin-induced alveolitis in the rat. *American Review of Respiratory Disease*.

[42] Zaman A., Cui Z., Foley J. P. (2005). Expression and role of the hyaluronan receptor RHAMM in inflammation after bleomycin injury. *American Journal of Respiratory Cell and Molecular Biology*.

[43] Yasuda T. (2007). Hyaluronan inhibits cytokine production by lipopolysaccharide-stimulated U937 macrophages through down-regulation of NF-*κ*B via ICAM-1. *Inflammation Research*.

[44] Neumann A., Schinzel R., Palm D., Riederer P., Münch G. (1999). High molecular weight hyaluronic acid inhibits advanced glycation endproduct-induced NF-*κ*B activation and cytokine expression. *FEBS Letters*.

[45] Park Y., Cho S., Linhardt R. J. (1997). Exploration of the action pattern of *Streptomyce*s hyaluronate lyase using high-resolution capillary electrophoresis. *Biochimica et Biophysica Acta (BBA)—Protein Structure and Molecular Enzymology*.

[46] Peshavariya H., Jiang F., Taylor C. J., Selemidis S., Chang C. W. T., Dusting G. J. (2009). Translation-Linked mRNA destabilization accompanying serum-induced Nox4 expression in human endothelial cells. *Antioxidants and Redox Signaling*.

