# Corrigendum to “Effect of High, Medium, and Low Molecular Weight Hyaluronan on Inflammation and Oxidative Stress in an *In Vitro* Model of Human Nasal Epithelial Cells”

**DOI:** 10.1155/2019/9198518

**Published:** 2019-10-03

**Authors:** Giusy Daniela Albano, Anna Bonanno, Luca Cavalieri, Eleonora Ingrassia, Caterina Di Sano, Loredana Riccobono, Rosalia Gagliardo, Mirella Profita

**Affiliations:** ^1^Institute of Biomedicine and Molecular Immunology “A. Monroy” (IBIM), National Research Council of Italy (CNR), Via Ugo La Malfa 153, 90146 Palermo, Italy; ^2^Chiesi Farmaceutici S.p.A, Parma, Italy

In the article titled “Effect of High, Medium, and Low Molecular Weight Hyaluronan on Inflammation and Oxidative Stress in an In Vitro Model of Human Nasal Epithelial Cells” [1], there was a duplication of bands and undeclared splicing in the Western Blots. After this was raised on PubPeer, the following concerns were confirmed:

- Figure 1(a) *β*-actin lanes 1 and 2 appear to be the same as Figure 4 *β*-actin lanes 1 and 2. They represent the same conditions (untreated cells and rh-IL-17A), but the duplication was not declared. Figure 1(a) *β*-actin lanes 1-3 are also the same as Figure 2 *β*-actin lanes 1-3, though the latter has been compressed horizontally to about 70% of the width. The third lane is not the same experiment.
- Figure 1(a) pERK lanes 1 and 2 are the same as Figure 3(b) pERK lanes 1 and 2, and they appear to represent the same conditions.
- Figure 1(b) *β*-actin lanes 1 and 2 are the same as Figure 3(b) *β*-actin lanes 1 and 2. Figure 1(b) pI*κ*B*α* lanes 1 and 2 are the same as Figure 3(b) pI*κ*B*α* lanes 1 and 2. However, the ratios reported in the respective bar charts above the blots are quite different.
- Figure 2(b) *β*-actin lanes 1 and 2 are the same as Figure 4(b) lanes 1 and 2, though the latter has been compressed horizontally to about 70% of the width.

The authors apologized for the confusion and said this was due to inadvertent human error. They prepared a new set of figures, which have no duplication. A declaration of splicing between the bands is included in the legends of Figures 1(a) and 2(b). The corrected figures, as approved by the editorial board, are as follows:

## Figures and Tables

**Figure 1:**
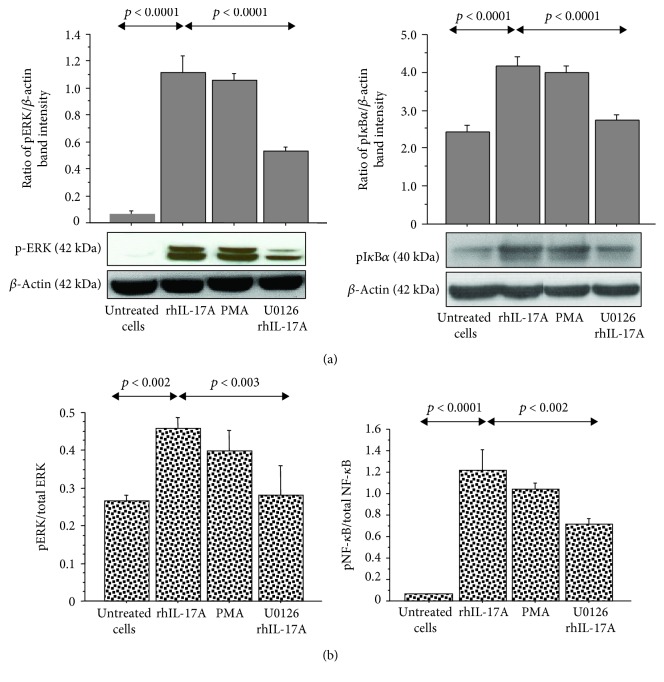
Effect of the U0126 inhibitor on ERK and I*κ*B*α* phosphorylation in RPMI 2650 cells stimulated with rhIL-17A. The cells were stimulated with rhIL-17A (20 ng/mL) or PMA (50 ng/mL) for 30 min in the absence or presence of U0126 (25 *μ*M). (a) pERK and pI*κ*B*α* protein expressions were evaluated in the cell lysates by western blot. The results were expressed as the ratio of band intensity and *β*-actin of 3 separate experiments. Representative gel images of pERK, pI*κ*B*α*, and *β*-actin are shown. The bands are spliced from uncropped blots. (b) The activation of ERK1/2 and NF-*κ*B for each experimental condition was tested for the pERK1/2/total ERK1/2 ratio and for the pNF-*κ*B/total NF-*κ*B, respectively, by ELISA, and normalized for protein content. ANOVA with Fisher's test correction was used for the analysis of the data. *p* < 0.05 was statistically significant.

**Figure 2:**
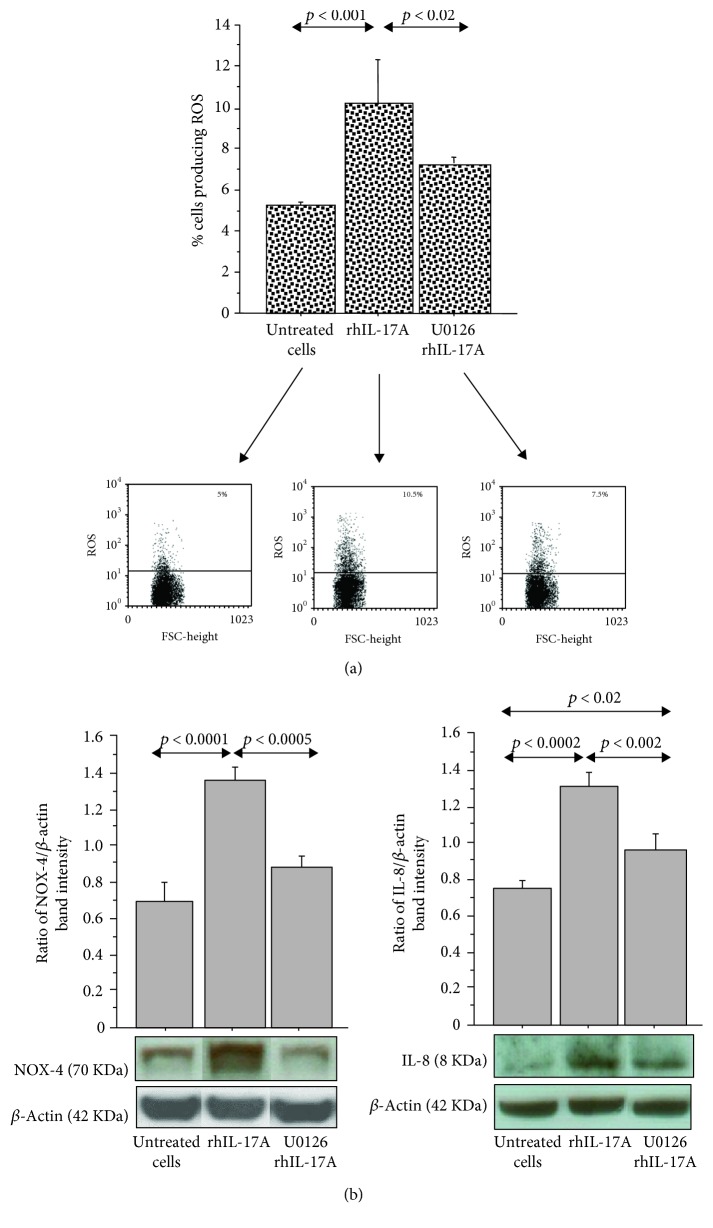
Effect of the U0126 inhibitor in RPMI 2650 cells stimulated with rhIL-17A. (a) The cells were stimulated with rhIL-17A (20 ng/mL) for 6 hrs in the absence or presence of U0126 (25 *μ*M). ROS production was evaluated in the cells by flow cytometry. The bars represent the mean ± SD of 3 separate experiments. Representative flow cytometry is shown; (b) the cells were stimulated with rhIL-17A (20 ng/mL) for 18 hrs in the absence or presence of U0126 (25 *μ*M). NOX-4 and IL-8 protein expressions were evaluated in the cell lysates by western blot. The results were expressed as the ratio of band intensity and *β*-actin of 3 separate experiments. Representative western blot is shown. The bands are spliced from uncropped blots. ANOVA with Fisher's test correction was used for the analysis of the data. *p* < 0.05 was statistically significant.

**Figure 3:**
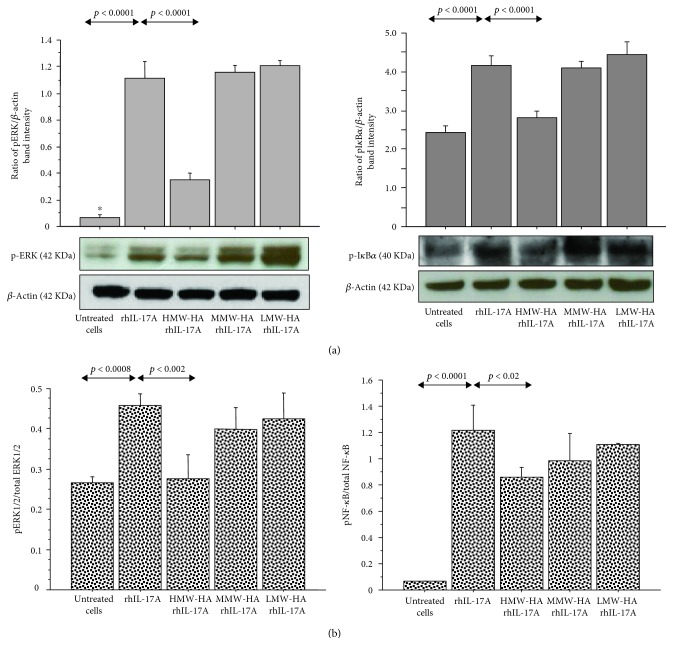
Effect of HMW-HA, MMW-HA, and LMW-HA on ERK1/2 and NF-*κ*B signal pathway in RPMI 2650 cells stimulated with rhIL-17A. The cells were preincubated with HMW-HA (100 *μ*g/mL), MMW-HA (100 *μ*g/mL), and LMW-HA (100 *μ*g/mL) for 1 h and then stimulated with rhIL-17A (20 ng/mL) for 30 min; (a) pERK and pI*κ*B*α* protein expressions were evaluated in the cell lysates by western blot. The bars represent the ratio of band intensity and *β*-actin of 3 separate experiments. Representative gel images of pERK, pI*κ*B*α*, and *β*-actin are shown; (b) the activation of ERK1/2 and NF-*κ*B for each experimental condition was tested for the pERK1/2/total ERK1/2 ratio and pNF-*κ*B/total NF-*κ*B ratio by ELISA and normalized for protein content. ANOVA with Fisher's test correction was used for the analysis of the data. *p* < 0.05 was statistically significant.

**Figure 4:**
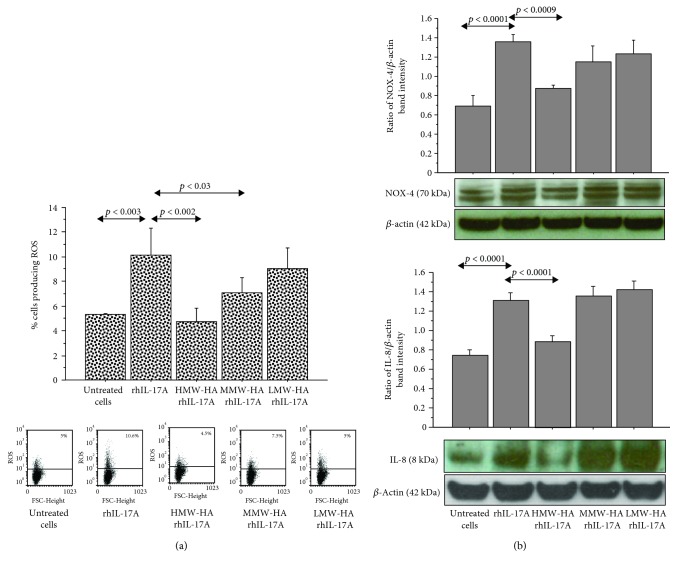
Effect of HMW-HA, MMW-HA, and LMW-HA in RPMI 2650 cells stimulated with rhIL-17A. (a) The cells were preincubated with HMW-HA (100 *μ*g/mL), MMW-HA (100 *μ*g/mL), and LMW-HA (100 *μ*g/mL) for 1 h and then stimulated with rhIL-17A (20 ng/mL) for 6 hrs. ROS production was evaluated in the cells by flow cytometry. The bars expressed the mean ± SD of 3 separate experiments. Representative flow cytometry is shown; (b) the cells were preincubated with HMW-HA (100 *μ*g/mL), MMW-HA (100 *μ*g/mL), and LMW-HA (100 *μ*g/mL) for 1 h and then stimulated with rhIL-17A (20 ng/mL) for 18 hrs. NOX-4 and IL-8 protein syntheses were evaluated in the cell lysates by western blot. The bars represent the ratio of band intensity and *β*-actin of 3 separate experiments. Representative western blot is shown. ANOVA with Fisher's test correction was used for the analysis of the data. *p* < 0.05 was statistically significant.

**Figure 5:**
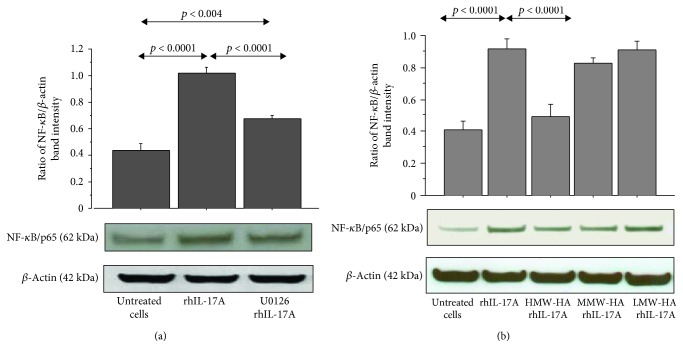
Effect of U0126, HMW-HA, MMW-HA, and LMW-HA on nuclear translocation of NF-*κ*B in RPMI 2650 cells stimulated with rhIL-17A. The cells were preincubated (a) with U0126 (25 *μ*M) or (b) with HMW-HA (100 *μ*g/mL), MMW-HA (100 *μ*g/mL), and LMW-HA (100 *μ*g/mL) for 1 h and then stimulated with rhIL-17A (20 ng/mL) for 30 min; NF-*κ*B was evaluated in nuclear cell lysate by western blot. The bars represent the ratio of band intensity and *β*-actin of 3 separate experiments. Representative gel images of NF-*κ*B and *β*-actin are shown. ANOVA with Fisher's test correction was used for the analysis of the data. *p* < 0.05 was statistically significant.

## Supplementary Materials

Supplementary Materials The uncropped gels showing the selected bands for Figures 1(a), 2(b), 3(a), and 4(b).
