# Supplementary material for: Corrigendum to “Effect of High, Medium, and Low Molecular Weight Hyaluronan on Inflammation and Oxidative Stress in an In Vitro Model of Human Nasal Epithelial Cells”
Source: Mediators Inflamm. 2019 Oct 3;2019:9198518. doi: 10.1155/2019/9198518 (PMC6794982; doi:10.1155/2019/9198518)
Supplement: Supplementary Materials — The uncropped gels showing the selected bands for Figures 1(a), 2(b), 3(a), and 4(b). [file 9198518.f1.pptx]

## Slide 1
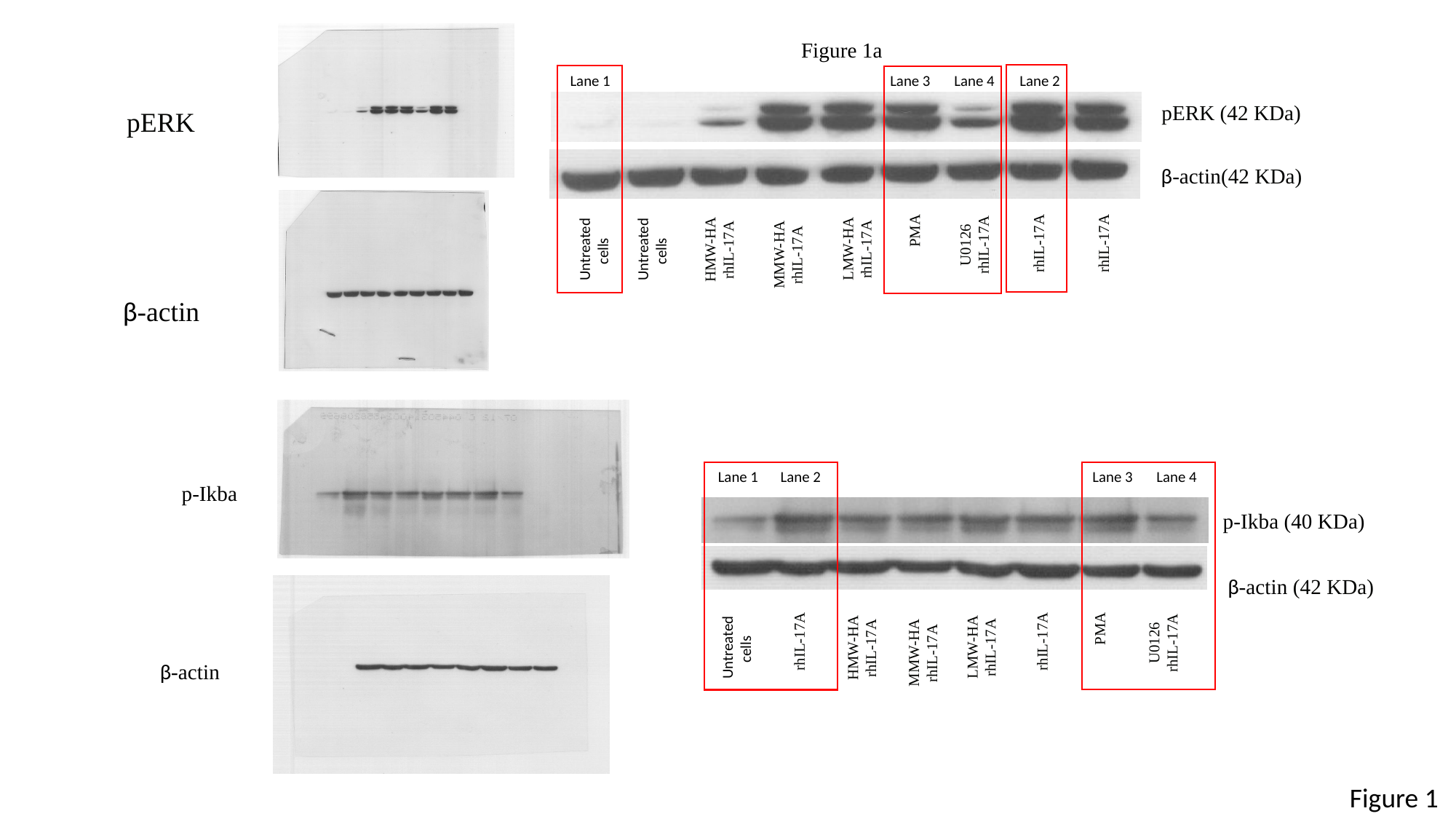

Figure 1a
pERK (42 KDa)
β-actin(42 KDa)
U0126
rhIL-17A
LMW-HA
rhIL-17A
HMW-HA
rhIL-17A
Untreated
cells
Untreated
cells
MMW-HA
rhIL-17A
PMA
rhIL-17A
rhIL-17A
Lane 1
Lane 3
Lane 4
Lane 2
pERK
β-actin
Lane 1
Lane 2
Lane 3
Lane 4
p-Ikba
p-Ikba (40 KDa)
β-actin (42 KDa)
U0126
rhIL-17A
LMW-HA
rhIL-17A
HMW-HA
rhIL-17A
Untreated
cells
rhIL-17A
PMA
rhIL-17A
MMW-HA
rhIL-17A
β-actin
Figure 1

## Slide 2
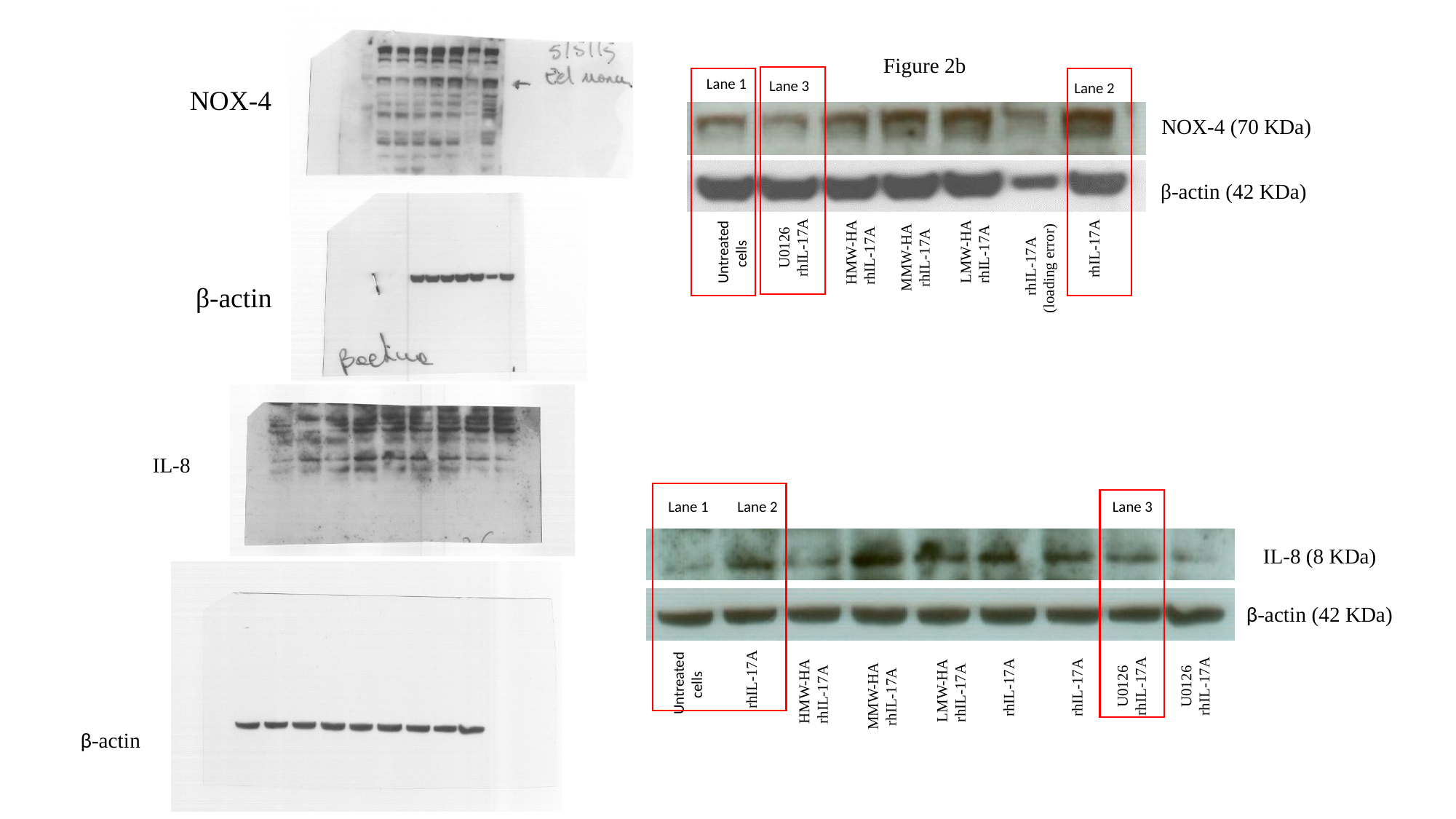

Figure 2b
Lane 1
Lane 3
Lane 2
NOX-4
NOX-4 (70 KDa)
β-actin (42 KDa)
U0126
rhIL-17A
LMW-HA
rhIL-17A
HMW-HA
rhIL-17A
Untreated
cells
MMW-HA
rhIL-17A
rhIL-17A
rhIL-17A (loading error)
β-actin
IL-8
Lane 1
Lane 2
Lane 3
IL-8 (8 KDa)
β-actin (42 KDa)
Untreated
cells
U0126
rhIL-17A
U0126
rhIL-17A
rhIL-17A
LMW-HA
rhIL-17A
HMW-HA
rhIL-17A
MMW-HA
rhIL-17A
rhIL-17A
rhIL-17A
β-actin

## Slide 3
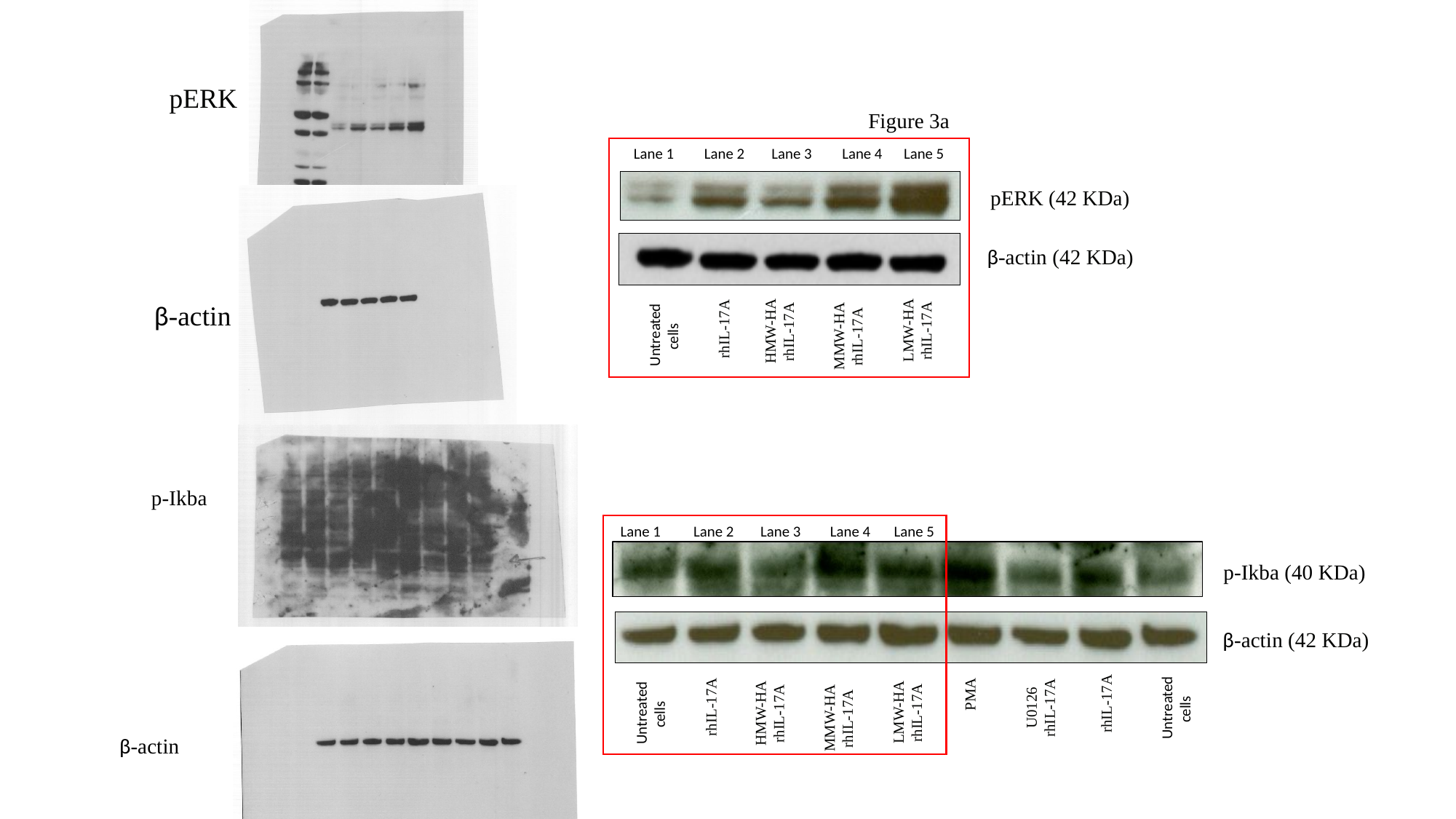

pERK
Figure 3a
Lane 1
Lane 2
Lane 3
Lane 4
Lane 5
pERK (42 KDa)
β-actin (42 KDa)
β-actin
LMW-HA
rhIL-17A
HMW-HA
rhIL-17A
Untreated
cells
MMW-HA
rhIL-17A
rhIL-17A
p-Ikba
Lane 1
Lane 2
Lane 3
Lane 4
Lane 5
p-Ikba (40 KDa)
β-actin (42 KDa)
LMW-HA
rhIL-17A
HMW-HA
rhIL-17A
Untreated
cells
MMW-HA
rhIL-17A
rhIL-17A
PMA
U0126
rhIL-17A
Untreated
cells
rhIL-17A
β-actin

## Slide 4
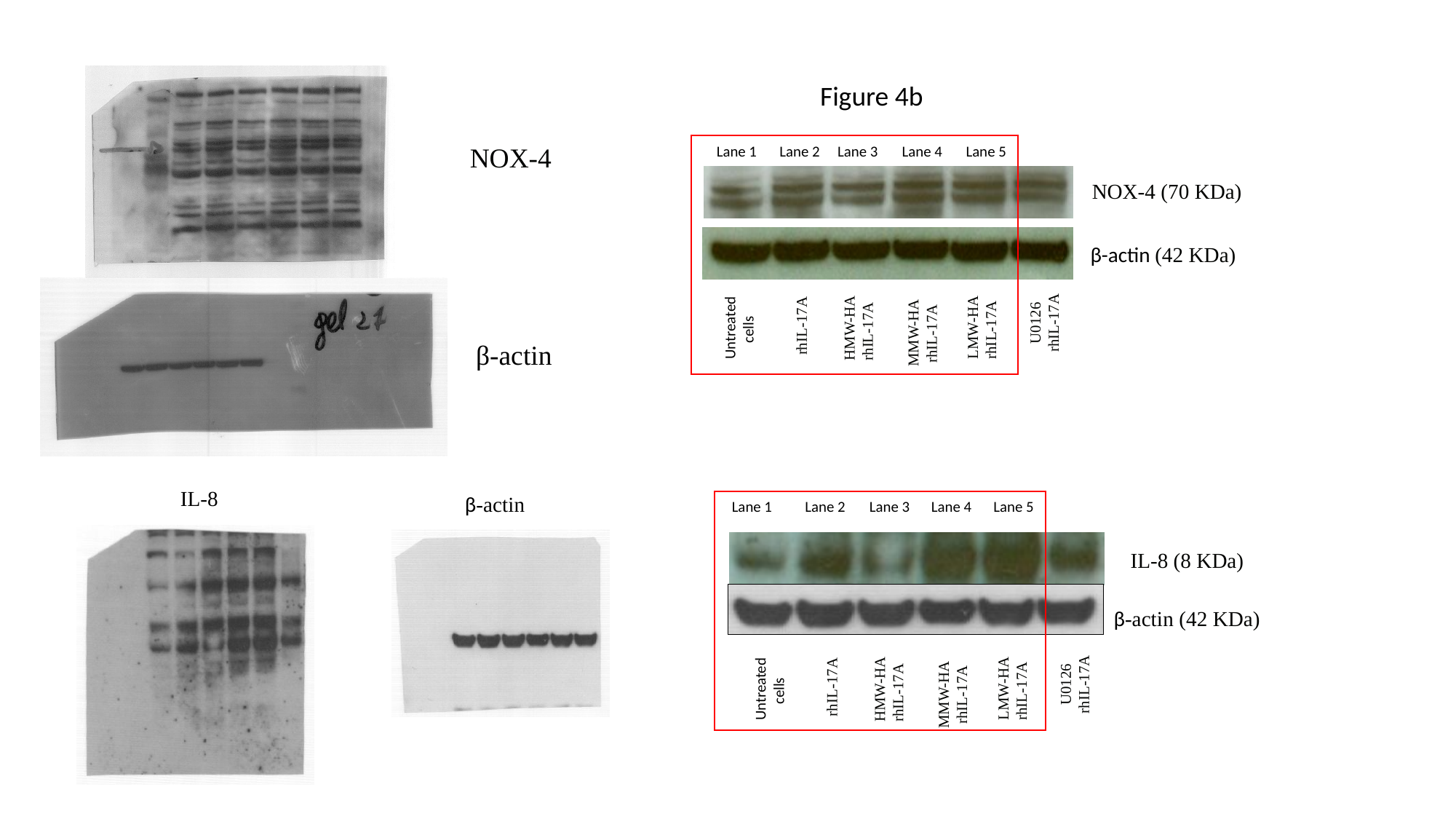

Figure 4b
NOX-4
Lane 1
Lane 2
Lane 3
Lane 4
Lane 5
NOX-4 (70 KDa)
β-actin (42 KDa)
U0126
rhIL-17A
LMW-HA
rhIL-17A
HMW-HA
rhIL-17A
Untreated
cells
MMW-HA
rhIL-17A
rhIL-17A
β-actin
IL-8
β-actin
Lane 1
Lane 2
Lane 3
Lane 4
Lane 5
IL-8 (8 KDa)
β-actin (42 KDa)
U0126
rhIL-17A
LMW-HA
rhIL-17A
HMW-HA
rhIL-17A
Untreated
cells
MMW-HA
rhIL-17A
rhIL-17A
